# Supplementary material for: The Distinctive Features behind the Aggressiveness of Oral and Cutaneous Squamous Cell Carcinomas
Source: Cancers (Basel). 2023 Jun 17;15(12):3227. doi: 10.3390/cancers15123227 (PMC10296732; doi:10.3390/cancers15123227)
Supplement: Supplementary file 1 [file cancers-15-03227-s001.zip › cancers-2421133-supplementary.pdf]

A: OSCC

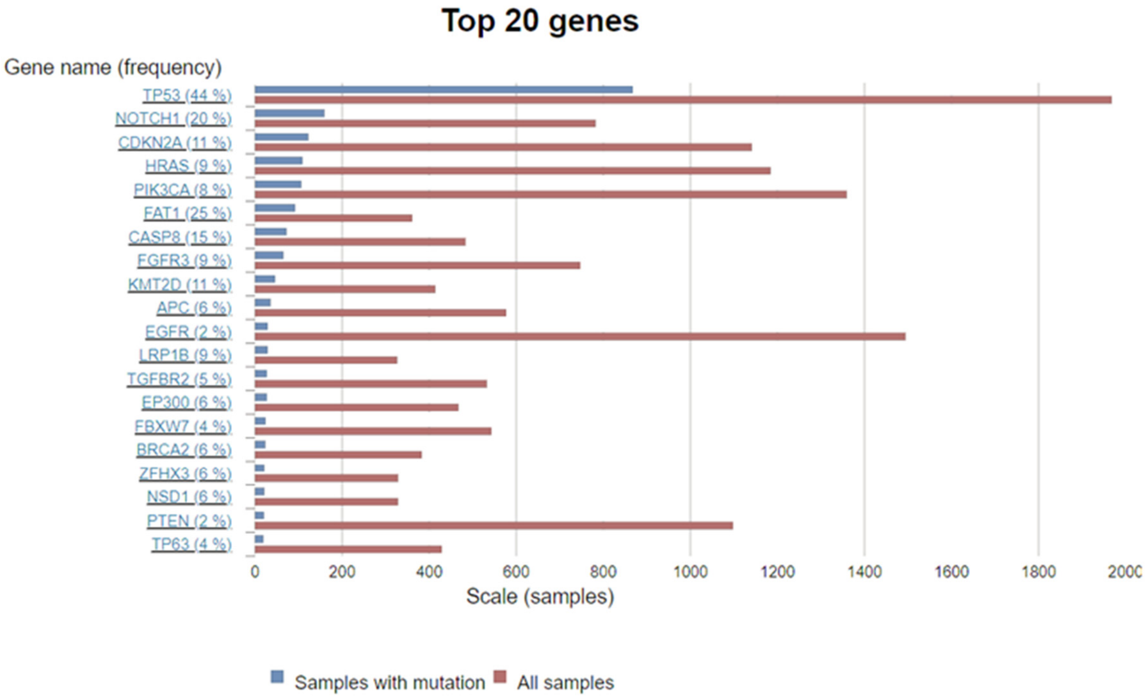

B: CSCC

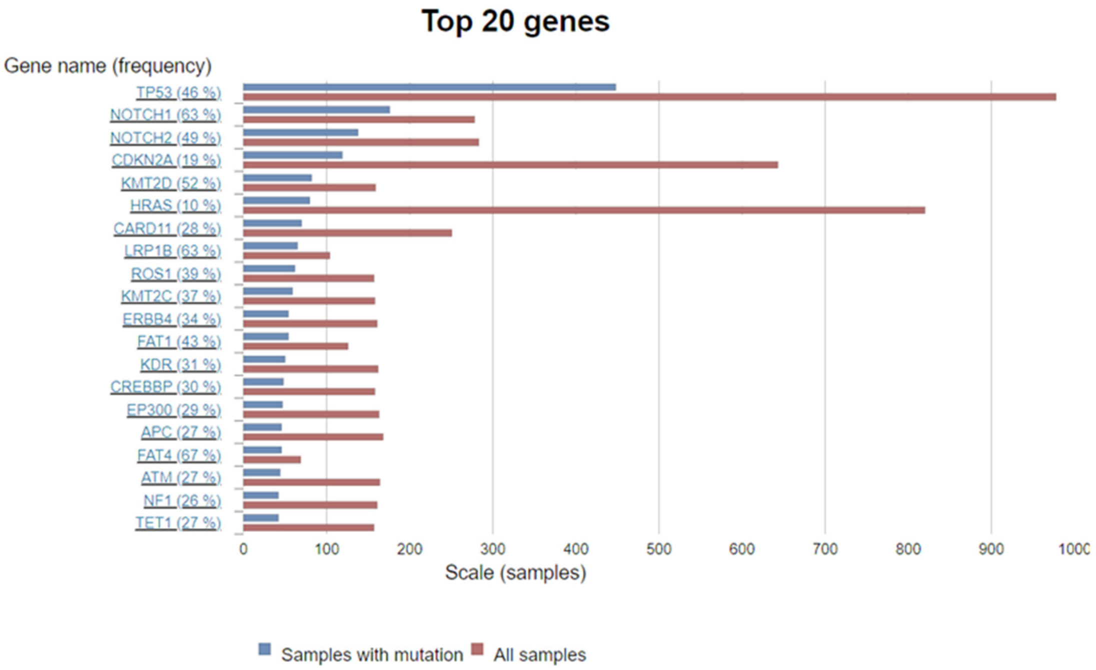

Supplementary Figure S1. Significantly top mutated genes in OSCC (A) and CSCC (B) in the COSMIC data base. In OSCC 1541 samples from upper aerodigestive tract´ tissue, mouth, squamous cell carcinoma are included (https://cancer.sanger.ac.uk/cosmic/browse/tissue?wgs=off&sn=upper_aerodigestive_tract&ss=mouth&hn=carcinoma&sh=squamous_cell_carcinoma&in=t&src=tissue&all_data=n). In CSCC, 677 of CSCC from skin, carcinoma, squamous cell carcinoma, are included (https://cancer.sanger.ac.uk/cosmic/browse/tissue?wgs=off&sn=skin&ss=all&hn=carcinoma&sh=squamous_cell_carcinoma&in=t&src=tissue&all_data=n).
